# Synthesis of carboxymethylcellulose/starch superabsorbent hydrogels by gamma-irradiation

**DOI:** 10.1186/s13065-017-0273-5

**Published:** 2017-05-30

**Authors:** Tamás Fekete, Judit Borsa, Erzsébet Takács, László Wojnárovits

**Affiliations:** 1 Institute for Energy Security and Environmental Safety, Centre for Energy Research, Hungarian Academy of Sciences, P.O. Box 49, Budapest 114, 1525 Hungary; 2 Faculty of Chemical Technology and Biotechnology, Budapest University of Technology and Economics, P.O. Box 91, Budapest, 1521 Hungary; 3 Faculty of Light Industry and Environmental Engineering, Obuda-University, Doberdó út 6, Budapest, 1034 Hungary

**Keywords:** Carboxymethylcellulose, Starch, Superabsorbent, Hydrogel, Irradiation, Crosslinking

## Abstract

**Background:**

Superabsorbent hydrogels show a large potential in a wide array of applications due to their unique properties. Carboxymethylcellulose (CMC) is a commercially available water-soluble cellulose derivative of major interest in the hydrogel synthesis. High-energy irradiation allows the chemical crosslinking without the use of crosslinking agents, while the introduction of other natural or synthetic polymers offers a convenient way to modify the gels. In this study we examined the effect of the addition of starch, a low-cost renewable polysaccharide, on the properties of carboxymethylcellulose-based hydrogels.

**Results:**

Superabsorbent gels were prepared by gamma irradiation from aqueous mixtures of carboxymethylcellulose and starch. The partial replacement of CMC with starch improved the gel fraction, while a slight increase in the water uptake was also observed. However, very high starch content had a negative impact on the gelation, resulting in a decrease in gel fraction. Moreover, higher solute concentrations were preferred for the gelation of CMC/starch than for pure CMC. Hydrogels containing 30% starch showed the best properties: a water uptake of ~350 g_water_/g_gel_ was achieved with ~55% gel fraction synthesized from 15 w/w% solutions at 20 kGy. Heterogeneous gel structure was observed: the starch granules and fragments were dispersed in the CMC matrix. The swelling of CMC/starch gels showed a high sensitivity to the ionic strength in water due to the CMC component. However, the mixed gels are less sensitive to the ionic strength than pure CMC gels.

**Conclusions:**

The introduction of starch to carboxymethylcellulose systems led to improved properties. Such gels showed higher water uptake, especially in an environment with high electrolyte concentration. CMC/starch hydrogels may offer a cheaper, superior alternative compared to pure cellulose derivative-based gels depending on the application.

## Background

Superabsorbent hydrogels are special materials capable of absorbing huge amount of water, usually more than 100 or even 1000 times of their dry weight, reaching much higher water content than conventional hydrogels [1]. The high absorbing capability and improved biocompatibility due to the high water content makes these hydrogels applicable in several fields. They are most commonly used in hygienic products, but their use as drug delivery systems [2], soil conditioners [3] and other non-hygienic applications [4, 5] is also becoming more and more important.

A wide array of polymers is used for superabsorbent production. Most commercial products are based on polyacrylates, but other synthetic polymers are also used, usually as copolymers with acrylates. However, there is a significant and ever growing interest in the use of natural materials for superabsorbent preparation. The focus of these studies is mainly on the most common and cheapest renewable resources, such as the cellulose [6], chitosan [7], starch and their derivatives, but other biomaterials like lignin [8] and various polysaccharide gums [9, 10] also show a large potential.

Cellulose is the most abundant renewable material in the world. However, due to its insolubility in water [11], for hydrogel formation there is a large interest toward its derivatives. Introducing various substituents in the cellulose structure decreases the number of the strong hydrogen bonds between the hydroxyl groups, thus water solubility can be reached relatively easily. Most commonly alkyl, hydroxyalkyl and carboxymethyl functional groups are used to modify cellulose [12]. For gelation purposes, carboxymethylcellulose (CMC) is in the center of research, but significant literature is available for other cellulose derivatives as well [6].

Carboxymethylcellulose-based hydrogels are prepared from aqueous solutions with several crosslinking methods. Crosslinking agents like polycarboxylic acids [13], epichlorohydrin [14] and *N*,*N′*-methylene-bisacrylamide (MBA) [15] are commonly used, but the gelation can also be achieved with multivalent cations like Fe^3+^ as well [16]. For the initiation of the crosslinking reaction in pure CMC high energy irradiation (both electron beam and gamma irradiation) is frequently applied [17]. A great advantage of irradiation is that gel formation occurs even without crosslinking agents. However, the presence of crosslinkers significantly improves the gelation process, resulting in better gelation and milder required synthesis conditions [18]. The gelation process is affected by several parameters, such as chemical structure and molecular mass of the polymer, solute concentration, absorbed dose [19] and atmosphere [20]. Radiation-initiated crosslinking was supposed to require high solute concentrations, as in dilute solutions the chain degradation processes are dominant [19]. However, recently gels were successfully synthesized from low concentration solutions at low pH, as well [21]. The swelling of the superabsorbents is usually sensitive to different environmental conditions, such as the temperature, pH, type of salt or ionic strength of the swelling solution [22, 23].

Starch is also a very cheap renewable resource, which is mostly used as a copolymer in synthetic polymer-based gels. Starch solutions are usually pregelatinized by heat treatment before the copolymerization to achieve a more homogeneous structure. Its free-radical crosslinking can be initiated either by initiator system [24, 25] or by high energy irradiation [26–28]. Such copolymer gels possess very high swelling capabilities. The gel properties are affected by several parameters, such as the starch source, which is related to the different amylopectin/amylose ratio [29].

Starch-based hydrogels combined with other renewable materials were not studied in-depth; while there is some literature available for starch/chitosan hydrogels [30], such gels have poor water uptake. Similarly, carboxymethylcellulose is mostly applied in copolymers with other cellulose derivatives [13, 31]; there is much smaller interest toward blends with other low cost, renewable materials [32, 33]. Cellulose and its water-soluble derivatives were used mostly for the preparation of various composite films with gelatinized starch [34–37]. Hydrogels were synthesized only with carboxymethylstarch and carboxymethylcellulose in the presence of MBA crosslinker [38]. Thus, there is no information available about the possible applicability of carboxymethylcellulose/starch blends for superabsorbent synthesis.

The goal of this work was to prepare cheaper CMC/starch hydrogels with improved superabsorbent properties as compared to pure CMC based gels. The gelation was achieved by gamma irradiation, without the use of crosslinking agents or other additives. The effect of the starch content on the gel properties at various synthesis conditions was examined. Moreover, the changes in various swelling properties such as the salt sensitivity with the blend ratio of the two components were also in-depth studied.

## Experimental

### Materials

Carboxymethylcellulose Na-salt (M_w_ = 700,000 g mol^−1^, D_s_ = 0.9, analytical grade), potato starch and NaCl (analytical grade) were purchased from Sigma-Aldrich and were used without purification.

### Synthesis

Carboxymethylcellulose and potato starch powder were mixed with blend ratios from 100:0 to 40:60. Solutions with solute concentrations ranging from 10 to 50 w/w% were prepared by adding deionized water to the blend. The presence of CMC provided a highly viscous, paste-like character, which made the dispersion of starch possible without a pregelatinization step. After stirring, the solution was stored for 24 h to achieve better homogeneity. From the homogenized material spherical samples with a mass of ~1 g were prepared. Samples were placed into polyethylene bags; the bags were closed and irradiated using ^60^Co γ-source—the crosslinking was carried out under air atmosphere. The absorbed dose ranged from 2.5 to 100 kGy at a dose rate of 9 kGy h^−1^. After irradiation, the gelled solutions were dried to constant weight at 60 °C.

### Gel fraction

Samples were immersed in deionized water to remove the sol fraction. A liquid:gel ratio of 1000:1 was used and the water was periodically changed. After 48 h the gel was removed by a metal sieve and dried to constant weight at 60 °C. The weight of the dry sample before (*w*_0_) and after (*w*_1_) the washing was used to determine the gel fraction:

1 $$Gel\,fraction\,\left ( \% \right) = \frac{{w_{1} }}{{w_{0} }} \times 100$$

### Degree of swelling

After the removal of the sol fraction the samples were dried and then immersed in deionized water at a liquid ratio of 1000:1. After 24 h the swollen gels were weighed and dried to constant weight at 60 °C (to recheck its weight due to the possible fragmentation of samples with very low mechanical stability). The weight of the swollen (*w*_*s*_) and the dry (*w*_*d*_) gel was used for the calculation of the degree of swelling:

2 $$Degree\,of\,swelling \left( {g_{water } \;g_{gel}^{ - 1} } \right) = \frac{{w_{s} - w_{d} }}{{w_{d} }}$$

The effect of the ionic strength was studied using NaCl solutions with concentrations from 0 to 0.2 mol dm^−3^.

### Gel composition

ATI Mattson Research Series FTIR spectrometer with ATR accessory (ZnSe flat plate, 45° nominal incident angle) was used to record the IR spectra of freeze-dried gel samples. The spectra were recorded at a resolution of 8 cm^−1^ from 4000 to 500 cm^−1^ with 128 scans; for the gel characterization the 2000–700 cm^−1^ range of the recorded spectra was used.

### Morphology

JSM 5600 V scanning electron microscope was used to study the morphology of the gels. Freeze-dried gels were used for sample preparation: they were cut and the cross-section was coated with gold. SEM images were recorded with 25 kV accelerating voltage at 35× to 1000× magnification.

## Results and discussion

### Synthesis parameters

The effect of three important synthesis parameters was studied in-depth: carboxymethylcellulose:starch ratio, solute concentration and absorbed dose.

#### Starch content

**Fig. 1:**
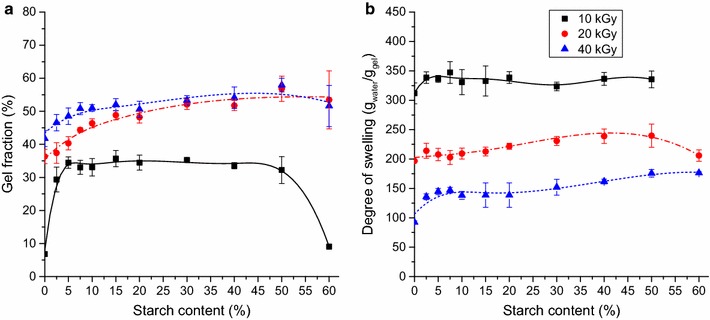
The effect of starch content on the gel fraction (**a**) and on the degree of swelling (**b**) of CMC/starch hydrogels (20 w/w% solution, absorbed doses: 10, 20 or 40 kGy)

The effect of starch content on the gel properties was studied at 10, 20 and 40 kGy absorbed dose. Pure CMC systems showed low gelation at 10 kGy, only a gel fraction of 7% was observed. At higher doses the gelation improved significantly and 35–40% gel fraction was reached. The replacement of 5–10% CMC with starch significantly increased the gel fraction at all doses (Fig. 1a). However, between 10 and 50% starch content the gel fraction did not change and at 10 kGy above 50% a sudden decrease was observed in the gel fraction. At 20 and 40 kGy above 60% this decrease was not observed, however, no gel formation was detected at 70% or higher starch content, including pure starch systems.

In aqueous solutions the radical processes are initiated mainly by the reactive intermediates (hydrated electron, OH radical and H atom) formed in the radiolysis of water. Below a certain solute concentration radiation induced direct chain scission is negligible. In the presence of dissolved oxygen the reactions of hydroxyl radicals should only be considered as the other two intermediates reacting with oxygen transform to the less reactive O_2_^•−^/HO_2_^•^ radical pair. In reactions of •OH with carbohydrates it abstracts an H-atom from a C–H bond with high yield [39, 40]. The carbon centered radicals formed will participate in both crosslinking and degradation reactions. In the case of cellulose and its derivatives the ratio of these two radical processes depends on the chemical structure, the solute concentration and on the degree of substitution. In 20% CMC solutions (D_s_ = 0.9) the crosslinking is the main process [23, 39]. In these circumstances both the mobility of the chains and the distance between the neighboring radicals are favorable for the reaction between two neighboring macroradicals leading to crosslink formation.

The starch granules also participate in the crosslinking process, leading to improved gelation: the CMC chains react with the granule surface through the recombination of the radicals formed on both polymers. The radical formation in the starch is similar to the reaction observed for the CMC due to the similar chemical structure. In this case the reaction is not hindered by electrostatic repulsion like during the crosslink formation between two CMC chains. The irradiation also affects the properties of the starch: the degradation processes lead to a decrease in the degree of polymerization, lower swelling and a more amorphous structure [41, 42]. This also increases the interaction between the CMC and starch due to the larger available granule surface. Moreover, with increasing starch ratio, the high viscosity caused mainly by the CMC became lower, thus the increased chain mobility also helped the gelation. At very high starch concentration the radiation degradable nature of starch prevails, besides CMC crosslinking is hindered by the large distance between the mobile CMC chains, leading to low or no gelation. Moreover, low doses lead to weaker crosslinking due to the lower number of radicals, thus the decrease in the gelation starts at lower starch content as seen at 10 kGy.

The swelling of pure CMC gels differed significantly depending on the adsorbed dose. At 10 kGy they exhibited a water uptake of ~300 g_water_/g_gel_ due to the poor gelation. Higher doses led to a major decrease in the swelling (~200 and ~100 g_water_/g_gel_ at 20 and 40 kGy, respectively), resulted by the higher crosslink density. Interestingly, similarly to the gel fraction, the water uptake also showed a small increase in the presence of starch (Fig. 1b). After an initial increase of ~50 g_water_/g_gel_ at 5% starch content, the degree of swelling showed no significant change at 40 kGy, but a small improvement (20–30 g_water_/g_gel_) was observed at high starch content using lower doses. The slight increase may be explained by the lower CMC content. Substituting CMC with starch has a similar effect as lowering the solute concentration, because the CMC concentration in the matrix is lower. At lower CMC concentration the water uptake increases due to the lower crosslink density in the CMC phase, which allows a larger expansion of the polymer network.

**Fig. 2:**
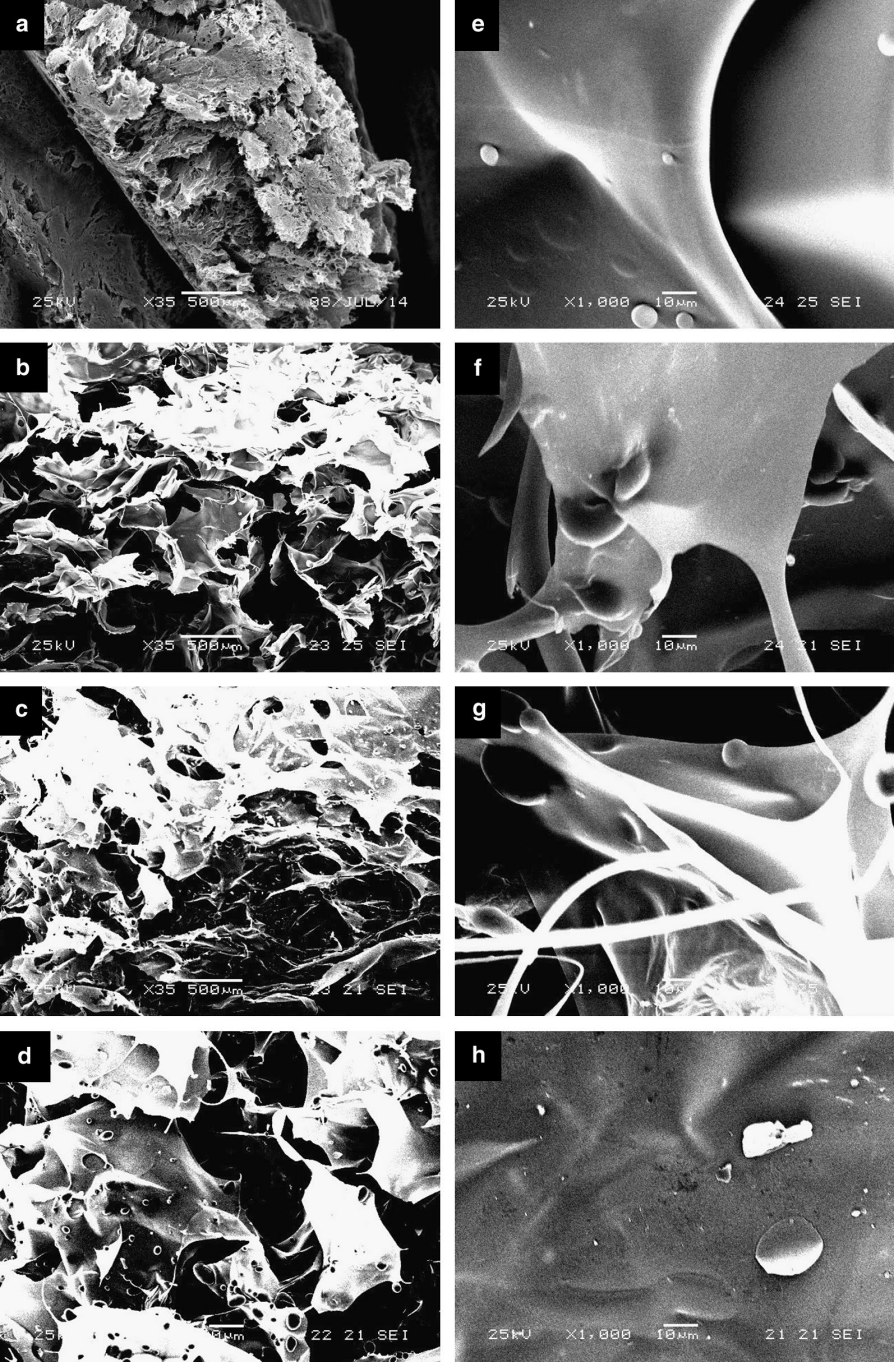
SEM photographs of freeze-dried CMC/starch hydrogels with a starch content of 0% **a**, 30% **b**, **e**–**h** and 50% **c**, **d** (×35 to ×1000 zoom; gel synthesis: 20 w/w% solution, 20 kGy dose)

The morphology of gels with different starch content was studied by SEM (Fig. 2). CMC/starch gels showed a highly porous structure like CMC gels (Fig. 2a–d). This is due to the high water content: the samples were freeze-dried after reaching the equilibrium water uptake, thus resulting in large pores. While the degree of swelling increased only slightly with the starch content, the pore size increased significantly compared to pure CMC gels. Presumably, the CMC network of CMC/starch gels is more flexible, thus larger expansion is possible, resulting in larger pore structure. This also explains the increase in the degree of swelling despite the very low water absorbing capacity of starch. The starch granules could be observed in the gel cross-section: some of them were on the surface of pores, while others were fully embedded in the CMC phase (Fig. 2e–h). The granules were distributed relatively evenly in the structure. With the increase of the starch content the density of the granules increased in the gel structure, thus the granules were properly linked to the CMC phase (Fig. 2c, d). The starch granules appeared mainly undamaged by the irradiation, though part of them were fragmented (Fig. 2h). According to the literature, the extent of the degradation observed depends on the environment, as well. While the irradiation of dry starch powder mainly modified the inner structure of the potato starch granules, their surface remaining visually unchanged in dry state [41]. However, in the presence of water fragmentation of the granules was observed even at low doses when starch was irradiated before the extraction from potato [43]. Thus, in our experiments the fragmentation can be explained by the high water content: the water radiolysis intermediates attack the starch molecules thus promoting the degradation. The partial fragmentation is advantageous as the radicals formed in inner part of the granules after fragmentation can also take part in the network formation.

**Fig. 3:**
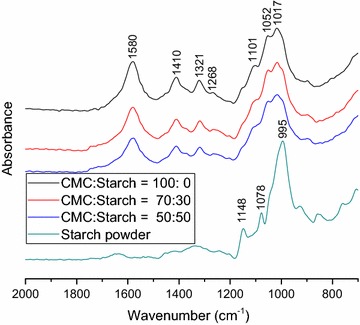
FTIR-ATR spectra of various freeze-dried CMC/starch gels (20 w/w%, 20 kGy) and starch powder

The gel composition of various CMC/starch gels was determined using FTIR-ATR (Fig. 3). The IR spectra were compared in the 500 and 2000 cm^−1^ wavenumber range. In case of CMC gels several characteristic peaks were observed [44]. An absorption band with multiple peaks in the 1150–1000 cm^−1^ range is attributed to the ether bonds in the cellulose backbone. The ionized carboxyl groups (COO^−^) show two absorption peaks at 1580 and 1410 cm^−1^ due to the symmetric and asymmetric stretching. Smaller peaks at 1321 and 1268 cm^−1^ can be assigned to the stretching vibrations at C=O and OH groups. In comparison, pure starch powder has a significantly different IR spectrum. Between 1150 and 1000 cm^−1^, similarly to the carboxymethylcellulose, peaks related to the COC stretching are observed [45]. However, a single high intensity peak appears at 995 cm^−1^ instead of the dual peak observed with 1017 and 1052 cm^−1^ for CMC. Low intensity bands at 1700–1600 cm^−1^ also appear, probably due to the water present in the amorphous phase.

In the IR spectra of CMC/starch gels, all the absorption peaks observed at pure CMC gels were also present. However, the intensities of the carboxyl absorption peaks became lower with increasing starch content, as starch does not contain carboxyl groups. As both polymers show a high absorption at 1150–1000 cm^−1^, the intensity of this band did not decrease. However, the peak at 1017 cm^−1^ became less sharp due to the absorption of starch at 995 cm^−1^. The change of the IR spectra shows the presence of both polymers in the gel, thus both components participate in the formation of the gel fraction.

#### Absorbed dose

**Fig. 4:**
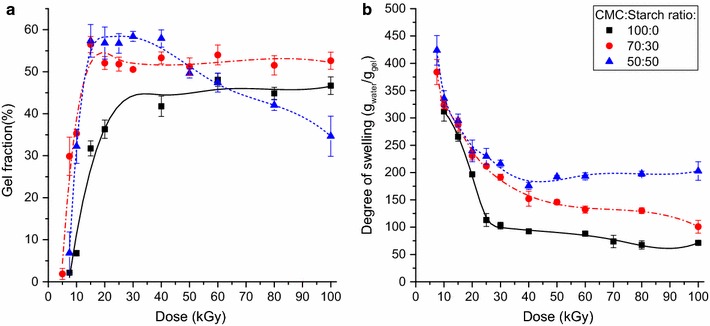
The effect of the absorbed dose on the gel fraction (**a**) and on the degree of swelling (**b**) of various CMC/starch gels (20 w/w% solution)

Based on previous results we concluded that the effect of absorbed dose on the gel properties should be investigated in more detail. It was studied at three different carboxymethylcellulose:starch ratios. For pure carboxymethylcellulose solutions, at doses lower than 8 kGy the formation of very loosely crosslinked systems with relatively low water uptake was observed (Fig. 4). The separation of the gel from the water by using sieve was not possible as such systems did not have sufficient mechanical stability and acted more like viscous liquids. The gel fraction increased with the dose up to 40 kGy (Fig. 4a) and water uptake decreased due to the higher crosslink density hindering the elongation of the polymer chains, thus reducing the water absorbing capacity (Fig. 4b). In pure CMC above this dose there was no further increase in gel fraction because the degradation became dominant. When increasing the starch ratio to 30 or 50%, the critical dose required for gelation decreased to 5 kGy, though acceptable gel fraction was reached only at 8–10 kGy in both cases. At higher doses the gel ratio increased by 10% compared to pure CMC gels and it remained practically constant (above 10 kGy) for samples containing 30% starch. For gels with 50% starch content the gel content started decreasing above 40 kGy showing the effect of degradation. In the 15–40 kGy dose range both starch containing samples showed similarly high degree of swelling and gel fraction. At 15 kGy the gel fraction was close to 60% and swelling degree about 300 g_water_/g_gel_. No significant change in gel content was observed up to 50 kGy while the swelling decreased constantly reaching 200 g_water_/g_gel_ for both gels at 40 kGy. For gels of 50% starch content no change in the swelling was observed, while for gels of 30% starch content the swelling ability slowly decreased, reaching 150 g_water_/g_gel_ at 100 kGy.

#### Solute concentration

**Fig. 5:**
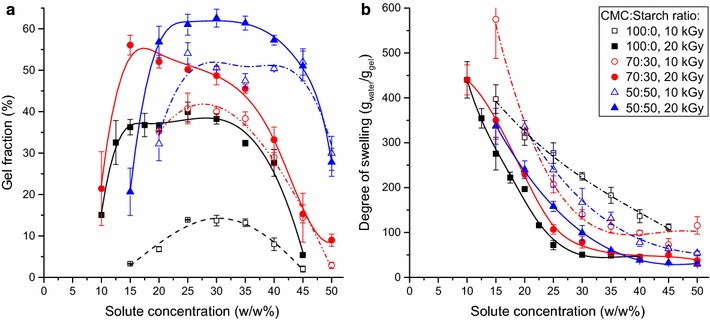
The effect of the solute concentration on the gel fraction (**a**) and on the degree of swelling (**b**) of various CMC/starch gels (absorbed dose: 10 or 20 kGy)

The effect of solute concentration was determined with samples irradiated with 10 and 20 kGy absorbed doses (Fig. 5). Very low and very high solute concentrations did not lead to gelation. This can be explained by the relatively large chain distance in the former case, resulting in the formation of a very loose physical network, thus the chain degradation becomes dominant compared to the crosslink formation. When the solute concentration is high, the crosslinking is hindered by the low polymer chain mobility due to the high viscosity of the solution. The gel fraction showed a plateau type maximum in a wide solute concentration range, but decreased with high slope under and over the critical concentration values. For pure CMC gels the highest gel fraction was observed in the 15–30 w/w% range at 20 kGy. Partially replacing CMC with starch led to a major increase in the gel ratio. The highest gel fraction was 50–55% at 30% starch content and 60% for gels with a CMC:starch ratio of 50:50, as compared to the 35–38% for pure CMC gels. The concentration range for maximum gel fraction also shifted to higher solute concentrations. Solutions with 50% starch content showed much lower gelation in lower solute concentrations.

The water uptake monotonously decreased with the solute concentration (Fig. 5b). This is related to the smaller polymer chain distance, which resulted in a more compact gel structure, thus the network expansion during the swelling was hindered. Replacing the CMC with starch led to a small increase in the degree of swelling, especially in the 20–30 w/w% concentration range. Increasing the starch content from 30 to 50% had only a minor impact on the water uptake at the 25–30 w/w% solute concentration range.

Lowering the dose to 10 kGy resulted in lower gel fraction but higher water uptake. Moreover, the critical solute concentration required for gelation and the maximum of the gel fraction shifted towards higher concentrations. CMC solutions at 10 kGy showed low gelation, the gel fraction being under 15% in the whole solute concentration range. While the gel fraction of CMC/starch gels also decreased due to the lower absorbed dose, over 20 w/w% it was still higher than for CMC gels synthesized at 20 kGy. At 20 w/w%, the gel fractions of CMC (20 kGy) and CMC/starch (10 kGy) gels were similar, but the latter had significantly higher water uptake. CMC solutions crosslinked at 10 kGy showed even higher swelling at higher solute concentrations due to the very weak network formation, but this also led to a very low gel fraction.

Based on the results, hydrogels containing 30% starch showed the best properties, as large improvement in the gelation was achieved with good swelling properties as compared to pure CMC based gels. Lowering the solute concentration proved to be more effective (having smaller impact on the gel fraction) in the improvement of the water uptake than changing the dose, the optimal properties requiring 15 w/w% solute concentration and 20 kGy dose. Such systems exhibited ~350 g_water_/g_gel_ water uptake and relatively high (~55%) gel fraction, significantly higher than observed for pure CMC hydrogels. Moreover, the swelling properties of these gels were higher than those of the carboxymethylcellulose-based superabsorbents with the same gel fraction prepared with crosslinking agent [18] or introducing low concentrations of acrylic acid [46]. On the other hand, CMC/starch systems needed higher solute concentration and dose to achieve the same gelation and showed inferior swelling properties at lower gel fractions. The use of starch allows avoiding the use of toxic monomers and crosslinkers, which may be a significant advantage depending on the application.

### Salt effect on swelling behavior

**Fig. 6:**
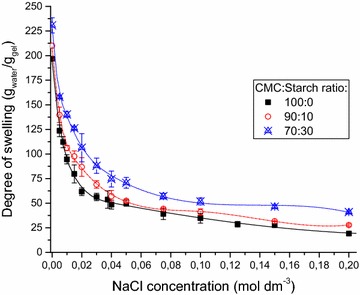
The effect of the NaCl concentration on the degree of swelling of various CMC/starch hydrogels (20 w/w% solution, 20 kGy)

The sensitivity to the ionic strength was determined with 0–0.2 mol dm^−3^ concentration NaCl solutions (Fig. 6). Pure CMC gels proved to be very sensitive to the NaCl concentration. The excellent swelling of CMC superabsorbents is related to the osmotic pressure of the Na^+^ cations and the improved elongation of chains due to the repulsion of charged carboxymethyl groups. The osmotic pressure decreases with the salt concentration, while the diffusion of the Na^+^ cations into the gel network shields the repulsion of the carboxymethyl groups. The effect of the salt concentration on water uptake of CMC/starch gels was lower than that observed for pure CMC gels, but they still showed high sensitivity. For example, the water uptake of CMC gels decreased by 82% at 0.1 mol dm^−3^ NaCl solution compared to the swelling in deionized water, while the decrease for CMC/starch gels was only 70–75%. It is important to note that the relative sensitivity to ionic strength increases with the equilibrium water uptake [18]. Yet, lower relative decrease in swelling was observed for CMC/starch gels despite the water uptake in deionized water being higher than that for pure CMC gels. Thus in various practical applications in environment with high ionic strength starch/CMC gels show much higher swelling than CMC gels.

## Conclusions

Hydrogels with superabsorbent properties were successfully prepared from carboxymethylcellulose/starch solutions. The addition of starch resulted in an increase both in the gel fraction and in the water uptake at relatively low doses. While starch alone is a radiation degradable polymer, in the presence of CMC the radicals formed on the starch chain will react with radicals on the CMC chain, leading to crosslinking instead of degradation. The partial replacement of the carboxymethylcellulose with starch up to a certain ratio offers an alternative to pure CMC gels with increased swelling in water. The optimal synthesis parameters proved to be 30% starch content, 15 w/w% solute concentration and 20 kGy absorbed dose. Such superabsorbent showed both high water uptake (~350 g_water_/g_gel_) and gel fraction (~55%), significantly higher than observed for pure CMC gels (200 g_water_/g_gel_ and 35%). Moreover, the presence of the starch also led to a lower sensitivity to the solvent properties such as the electrolyte content. While responsive behavior is crucial for several applications, in certain fields such as the agriculture only the very high water absorption capacity is utilized. In such conditions the application of carboxymethylcellulose/starch systems, which exhibit good swelling properties but lower sensitivity to the presence of salts or the pH of the soil, may be favored to pure polyelectrolyte-based superabsorbents.

## Abbreviations

CMC: carboxymethylcellulose
FTIR: Fourier transform infrared spectroscopy
ATR: attenuated total reflectance
SEM: scanning electron microscopy
